# Natural Alkaloids as Antimicrobial Agents: Mechanisms, Potentials and Challenges

**DOI:** 10.3390/molecules31071204

**Published:** 2026-04-05

**Authors:** Xi-Zhong Zhang, Ming-Xia Chen, Rui Hou, Wan-Qin Wang, Zhen-Dan He, Jie-Shu You, Xun Song

**Affiliations:** 1 College of Pharmacy, Shenzhen Technology University, Shenzhen 518118, China; 2310415078@stumail.sztu.edu.cn (X.-Z.Z.); 2210415048@stumail.sztu.edu.cn (M.-X.C.); hezhendan@sztu.edu.cn (Z.-D.H.); 2 School of Pharmaceutical Sciences, Shenzhen University, Shenzhen 518000, China; 3 Nam Yue Natural Medicine Co., Ltd., Macau, China; hour@nynature.vip (R.H.); wangwq@nynature.vip (W.-Q.W.)

**Keywords:** antimicrobial resistance, alkaloids, antibacterial, antifungal, antiviral, drug discovery

## Abstract

Antimicrobial resistance (AMR) poses a significant global health threat, with multidrug-resistant pathogens undermining the effectiveness of conventional antibiotics. Natural alkaloids, a diverse group of nitrogen-containing compounds mainly derived from plants, are gaining attention as potential antimicrobial agents due to their broad-spectrum activity, structural variety, and unique mechanisms of action. This review examines the antimicrobial properties of natural alkaloids, classifying them by chemical structure (e.g., quinoline, isoquinoline, pyridine, indole, and imidazole alkaloids). Their antibacterial, antifungal, and antiviral activities are discussed, along with the mechanisms by which they target pathogenic microorganisms, including disruption of cell walls and membranes, inhibition of protein synthesis, interference with DNA replication, and viral assembly. The review also explores the synergistic effects of alkaloids when combined with conventional antimicrobial agents. Alkaloids demonstrate potent antimicrobial activity against various pathogens. Quinoline alkaloids, such as quinine, inhibit DNA replication and damage cell membranes. Isoquinoline alkaloids like berberine and sanguinarine exhibit broad-spectrum antibacterial effects. Pyridine alkaloids, including nicotine, disrupt bacterial membranes. In fungi, alkaloids such as sanguinarine and indole derivatives prevent cell wall synthesis and spore germination. Antiviral alkaloids like lycorine target viral RNA polymerases. Additionally, alkaloids enhance the activity of traditional antibiotics by overcoming resistance. Natural alkaloids represent a promising source of antimicrobial agents with diverse mechanisms to combat AMR. Future research should focus on optimizing alkaloid structures, ensuring safety and efficacy, and exploring combination therapies to address the escalating AMR challenge.

## 1. Introduction

The World Health Organization (WHO) has identified antimicrobial drug resistance (AMR) as one of the ten most significant global public health threats [1]. This growing issue encompasses not only bacteria [2] but also fungi [3], viruses, and other pathogens. The dissemination of AMR has resulted in elevated healthcare expenses, prolonged treatment periods, and, in critical instances, heightened mortality rates. The rise and spread of AMR have resulted in rising healthcare costs, extended treatment periods, and, in severe cases, higher mortality rates. Specifically, the emergence of multidrug-resistant and pan-drug-resistant bacteria has led to a scenario where many infections can no longer be effectively treated with existing antimicrobial agents, including antibiotics [4]. Current research forecasts that by 2050, AMR might cause 10 million deaths each year globally, accompanied by substantial economic impacts [5]. In only 80 years, the antibiotic era has seen the widespread rise of drug-resistant pathogens [6]. In response, the Chinese government launched the China Action Plan to Combat Bacterial Resistance (2016–2020), targeting the reduction of irrational antimicrobial drug use and addressing the AMR crisis [7]. The emergence and spread of drug-resistant pathogens pose a global challenge requiring coordinated efforts from governments and the international community, bolstered by multidisciplinary collaboration and comprehensive strategies [8]. Additionally, creating new antimicrobial agents and alternative therapies is essential to addressing this problem. Only through ongoing global collaboration can we effectively tackle the threats from drug-resistant pathogens and protect human health.

Alkaloids, including pyridine, indole, and quinoline derivatives, are nitrogen-containing heterocyclic compounds primarily derived from plant sources [9]. They represent one of the most important classes of natural products, owing to their abundance, structural diversity, and complexity. Many alkaloids exhibit notable pharmacological activities, such as antibacterial [10], anti-inflammatory [11], anti-cancer [12], antiviral [13], analgesic, and neuroprotective effects [14]. For instance, the morphine-like alkaloid milrinone demonstrates both anti-inflammatory and pain-relieving properties [15], while quinine, derived from the bark of the cinchona tree, has long been used as an antimalarial agent [16]. Additionally, numerous alkaloids have been developed into modern pharmaceuticals for treating various diseases [17,18].

The antimicrobial mechanisms of alkaloids are diverse, encompassing inhibition of fungal cell wall synthesis [19], interference with DNA replication [20], disruption of protein synthesis [21], damage to bacterial cell membranes [22], and disruption of bacterial metabolic pathways [23]. Alkaloids offer promising new approaches for the development of antimicrobial agents. As natural antibiotics, they are sourced from a variety of natural resources and demonstrate potent antimicrobial activity against a wide range of clinical pathogens, including drug-resistant strains. This review provides an overview of the origin, chemical structure, antimicrobial activity, mechanisms of action, and potential synergistic effects of alkaloids with conventional antimicrobials. The goal is to contribute to strategies for combating bacterial resistance and offer insights for the development of new antibacterial therapies.

## 2. Overview of Alkaloids

Alkaloids have been the subject of extensive research as potential therapeutic agents since the discovery of Narcan in 1803 and the isolation of morphine in 1806 [24]. However, the use of alkaloid-containing substances dates back to ancient Greece, where poppy seed preparations were utilized for pain relief [25]. In 1983, Pelletier proposed that alkaloids are cyclic compounds containing nitrogen atoms that are typically oxidized, and are primarily found in more evolutionarily advanced plant species. These compounds often exhibit biological activity, serving as natural defense mechanisms for plants, and they possess significant medicinal properties for humans, exemplified by substances such as quinine [26], atropine [27], and ephedrine [28]. Alkaloids display a remarkable range of structural diversity, from simple compounds like imidazoles and pyridines to more complex structures such as terpenoid alkaloids.

In this review, the databases searched include PubMed, Web of Science, Scopus, and the Cochrane Library, covering publications from 2000 to 2025. Keywords such as alkaloids, antimicrobial, antibacterial, antifungal, antiviral, and natural products were used. Both *in vitro*, *in vivo*, *in silico* studies, and review articles were considered. The following section (Table 1) will explore the various typical chemical structures and rich natural sources of alkaloids.

## 3. Antimicrobial Effects of Alkaloids

### 3.1. Antibacterial Activity of Alkaloids

Alkaloids exhibit potent broad-spectrum antibacterial activity against both Gram-positive and Gram-negative bacteria via multifaceted mechanisms. Predominantly, these compounds compromise bacterial cell wall and membrane integrity, thereby disrupting intracellular homeostasis and inducing cell death [9]. Additionally, they inhibit essential cellular processes, including protein synthesis, DNA replication, and critical enzymatic functions within metabolic pathways [69]. The remarkable antimicrobial efficacy of natural alkaloids and their derivatives underscores their significant potential as therapeutic agents against bacterial pathogens.

#### 3.1.1. Examples of Alkaloids Exhibiting Antibacterial Activity

Quinoline alkaloids are a class of alkaloids defined by the presence of a quinoline ring in their chemical structure. These compounds are extensively found in nature, present in plants, microorganisms, and marine organisms [70]. Owing to their varied pharmacological characteristics, quinoline alkaloids have attracted considerable research attention, especially for their antibacterial effects. A well-known example is quinine, extracted from the bark of the cinchona tree, which has been used for a long time in treating malaria, a disease caused by the Plasmodium parasite and spread by infected mosquitoes. Quinine disrupts bacterial DNA replication and protein synthesis, resulting in bacterial cell death [71]. Quinidine, a compound with a structure akin to quinine, also exhibits antibacterial properties [72]. Although mainly used as an antiarrhythmic drug to treat irregular heartbeats, quinidine has been investigated for its antibacterial effects. Research shows that quinidine compromises bacterial cell membrane integrity, thus inhibiting the growth of specific bacterial strains [72]. It is suggested that further investigation into the structure-activity relationships (SAR) and optimization of quinoline alkaloids could lead to the discovery of new antibacterial drugs with enhanced efficacy and improved safety profiles [73].

Isoquinoline alkaloids represent a varied class of compounds defined by the presence of an isoquinoline ring within their chemical framework. These alkaloids are commonly found in nature, present in diverse plants and microorganisms. Owing to their unique structure and bioactivity, isoquinoline alkaloids have attracted considerable attention, especially for their antibacterial effects. A representative example is berberine, an isoquinoline alkaloid derived from plants of the Berberidaceae family. These plants have long been used in traditional herbal medicine, particularly in folk remedies for the treatment of bacterial infections such as gastrointestinal disorders and wound infections. This historical use highlights their empirical antimicrobial value and supports the continued investigation of berberine as a bioactive compound with therapeutic potential [74]. Berberine exhibits strong inhibitory effects against a wide array of both Gram-positive and Gram-negative bacteria. Its antibacterial action is thought to involve disrupting bacterial cell wall integrity and interfering with essential metabolic processes [75]. Another significant isoquinoline alkaloid is sanguinarine, which is also employed in traditional medicine. Sanguinarine shows antibacterial effects against various bacterial strains, including both Gram-positive and Gram-negative species. Its mechanism of action probably involves disrupting bacterial cell membranes and inhibiting key metabolic pathways [76]. Isoquinoline alkaloids hold considerable promise for the development of next-generation antibacterial therapies. Further investigation is needed to fully elucidate their mechanisms of action and assess their therapeutic potential in the treatment of bacterial infections. The discovery of these compounds offers valuable prospects for the design and development of novel antibacterial agents.

Pyridine alkaloids are a class of compounds characterized by the presence of a pyridine ring in their chemical structure. These naturally occurring substances are found in a wide variety of plant species, with a notable prevalence in the Solanaceae family. Pyridine alkaloids have attracted significant attention due to their diverse biological activities, including their potential as antibacterial agents [69]. Nicotine, a well-known pyridine alkaloid found in abundance in tobacco (*Nicotiana tabacum*), has been extensively investigated for its antibacterial properties. Historically, *N. tabacum* has also been used in traditional and folk medicine for its antimicrobial effects, including the treatment of skin infections and wounds, reflecting its early empirical use as a natural antibacterial agent. Studies have shown that nicotine exerts inhibitory effects against certain Gram-positive bacteria, such as *Staphylococcus aureus* [77]. Its antibacterial mechanism likely involves disruption of bacterial cell membranes and interference with critical metabolic processes. Another important pyridine alkaloid with antibacterial potential is anabasine, which is predominantly found in plants of the *Nicotiana* genus, particularly *N. glauca*. Research has demonstrated that anabasine exhibits antibacterial activity against both Gram-positive and Gram-negative bacterial species. Its mode of action includes the disruption of bacterial membrane integrity and the inhibition of bacterial enzymatic functions [78].

In addition to nicotine and anabasine, ongoing research continues to explore other pyridine alkaloids for their antibacterial properties. These studies highlight the potential of pyridine alkaloids as a promising foundation for the development of novel antibacterial agents. Further investigation into their mechanisms of action and therapeutic applications is necessary to fully assess their efficacy in combating bacterial infections.

#### 3.1.2. Mechanisms of Action Against Bacterial Pathogens

**Figure 1:**
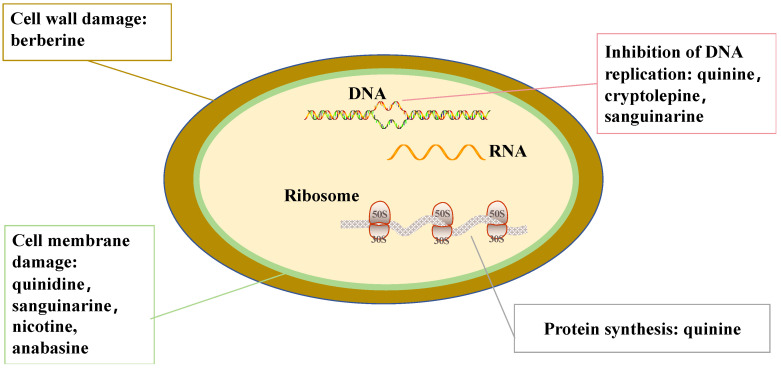
Representative antibacterial targets of natural alkaloids.

In recent years, there has been a marked rise in research focusing on the antibacterial mechanisms of natural alkaloids. As shown in Figure 1, these compounds have been shown to impede bacterial proliferation through various biological pathways, including disruption of the bacterial cell wall, inhibition of protein biosynthesis, and interference with DNA replication processes.

##### Disruption of Bacterial Cell Walls and Membranes

In the field of microbiology, it is crucial to acknowledge the essential functions of the cell wall and cytoplasmic membrane in ensuring the survival of bacteria. These structural elements are of great significance as they act as a barrier, preventing the escape of intracellular components and thus safeguarding the integrity of the bacterial cell [79]. The semi-permeable cytoplasmic membranes, consisting of phospholipid bilayers and protein complexes, create a stable internal milieu that is favorable for the normal physiological activities of bacterial life. When the cell wall fails to fulfill its protective role, it inevitably results in the disruption of the membrane’s transport and signaling pathways [80].

The impact of natural alkaloids on bacterial cell membranes and cell walls has been well-documented. Berberine has been shown to compromise the integrity of bacterial cell membranes and inhibit the formation of multidrug-resistant *Escherichia coli* [81]. A similar effect was observed with sanguinarine in the inhibition of *Providencia rettgeri*, where treatment at the minimum inhibitory concentration (3.9 μg/mL) led to disruption of cell membrane integrity, accompanied by leakage of intracellular ATP [76]. Alkaline phosphatase (AKP), primarily localized in the bacterial cell wall and membrane, can be released when cell wall permeability is increased [82]. Gu *et al.* reported that treatment with sanguinarine resulted in elevated extracellular AKP levels in *S. aureus*, suggesting that its antibacterial action may primarily target the bacterial cell wall.

##### Interference with Protein Synthesis

Proteins are essential components of bacterial life and play a central role in cellular processes. Consequently, alterations in protein levels can significantly impact the biological functions within bacterial cells [83]. *Holarrhena antidysenterica* has traditionally been used in folk medicine to treat bacterial infections, particularly gastrointestinal diseases such as dysentery and diarrhea, reflecting its longstanding antimicrobial application [84]. Alkaloids derived from the ethanolic extract of *H. altidyselllerica* seeds have demonstrated notable antibacterial activity against clinical isolates of pathogenic *E. coli*, with observed inhibition of high molecular weight protein synthesis [85]. Furthermore, research suggests that indole alkaloids may impair various protein and enzyme activities in bacteria, thereby contributing to their antibacterial properties [86].

##### Inhibition of DNA Replication

The primary function of DNA molecules is to store, replicate, and transmit genetic information. Therefore, damage to DNA or inhibition of its replication can prevent the expression of bacterial virulence genes, disrupting the normal growth and reproduction of microorganisms [87]. Several alkaloids, such as sanguinarine and cryptolepine hydrochloride, have been shown to interact with DNA, forming crosslinks that inhibit both DNA replication and transcription [88,89]. Additionally, studies indicate that berberine exhibits *in vitro* anti-invasive properties in *Galleria mellonella* infection models [80]. By influencing membrane potential and promoting intracellular accumulation, berberine acts as a DNA intercalator, binding to DNA and RNA molecules, thereby altering their structure and potentially disrupting their integrity. This interference hampers bacterial DNA replication, RNA transcription, and protein synthesis, contributing to its antimicrobial activity [90,91].

### 3.2. Antifungal Activity of Alkaloids

#### 3.2.1. Examples of Alkaloids with Antifungal Activity

In recent years, the spread of antifungal drug resistance has become increasingly prominent, which is closely associated with the rising morbidity and mortality of fungal infections. Since the molecular mechanisms of humans and fungi are highly similar, fungicidal compounds often pose risks of toxicity to host cells, making the search for natural antifungal agents particularly urgent. Natural alkaloids have shown significant antifungal activity. Isoquinoline alkaloids, commonly known as benzylisoquinoline alkaloids, are a well-studied class of plant secondary metabolites with an isoquinoline backbone, and their antifungal properties have been confirmed by numerous studies [92]. Indole alkaloids, derived from tryptophan, possess complex chemical structures and diverse biological activities [86], including notable antifungal effects. Imidazole alkaloids also exhibit promising antifungal potential: for example, platydesmine, an imidazole alkaloid extracted from *Pilocarpus grandiflorus*, a plant traditionally used in folk medicine for treating microbial infections, has been found to exert antifungal activity against *Leucoagaricus gongylophorus* [93].

#### 3.2.2. Mechanisms of Action Against Fungi

##### Destruction of Fungal Cell Walls

**Figure 2:**
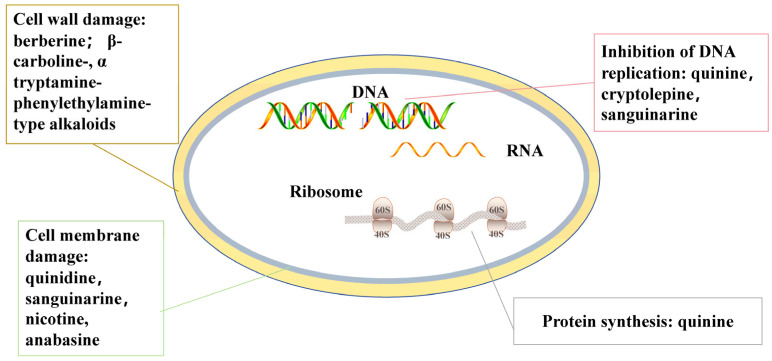
Mechanism of action of antifungal natural alkaloids along with their target sites.

The fungal cell wall is a primary target of natural alkaloids. Sanguinarine, for instance, has demonstrated antifungal activity against *Magnaporthe oryzae*. Treatment with sanguinarine at 10 μg/mL resulted in significant morphological changes in *M. oryzae*, including damage to the plasma membrane integrity and alterations in cell wall permeability (Figure 2). Additionally, leakage of intracellular contents and changes in mycelial structure were observed [94]. Recently, a novel alkaloid isolated from *Cyathobasis fruticulosa* has shown activity against *Aspergillus* and *Candida* species. *In vitro* testing revealed that this compound adversely affects the fungal cell wall [95,96].

##### Inhibition of Fungal Growth

Indole alkaloids, such as Kopsiafrutine C, D and E, isolated from *Kopsia fruticosa* exhibited promising antifungal activity against *C. albicans*, *C. glabrata*, and *C. tropicalis*, with minimum inhibitory concentrations (MICs) ranging from 0.3 to 1.14 mM [97]. Similarly, two indole alkaloids isolated from *Bacillus thuringiensis* and *Bacillus velezensis* strains inhibited the growth of 60–85% of fungi after 48 h of exposure to *Streptococcus* and *Moniliophthora roreri* [98]. Furthermore, Hassan *et al.* isolated an imidazole alkaloid, Naamine G, from the Indonesian sponge *Leucetta chagosensis*. This compound demonstrated significant antifungal activity against the plant pathogenic fungus *Cladosporium herbarum* [99].

##### Inhibition of Fungal Spore Germination

Fungal spores are essential to the life cycle of fungi, serving as reproductive units responsible for both reproduction and dispersal, thus ensuring the survival of fungal populations. Under favorable environmental conditions, spores can germinate to form new mycelium, marking the beginning of a new stage in the fungal life cycle. Sanguinarine has been shown to inhibit the development of *Magnaporthe oryzae*, with complete suppression of spore germination at a concentration of 50 μg/mL [94]. The addition of venenatin to an acetic acid solution effectively inhibited spore germination in ten different fungal strains. Notably, *Fusarium udum*, *Alternaria brassicicola*, *Ustilago cynodontis*, and *Aspergillus flavus* were particularly sensitive to this compound, with spore germination levels remaining below 10%. The parasitic fungus *Erysiphe pisi*, responsible for powdery mildew on *Pisum sativum*, was also significantly affected, with marked inhibition of both spore germination and colony development on excised leaves. Furthermore, pretreatment with venenatin had a greater impact on spore germination and colony formation than post-inoculation treatment [100].

### 3.3. Activity of Alkaloids Against Virus

#### 3.3.1. Examples of Alkaloids with Antiviral Activity

Plants and animals are estimated to harbor a wide variety of viruses, many of which are transmitted by insects. Evaluating antiviral activity is challenging, but several studies have investigated the effects of natural alkaloids on viral activity. Approximately 40 alkaloids, including quinoline and purine alkaloids, have demonstrated antiviral properties. Quinoline alkaloids, derived from the tropical vine *Ancistrocladus korupensis*, have shown inhibitory effects against HIV [101]. Additionally, various isoquinoline alkaloids isolated from the Amaryllidaceae family, such as lycorine, narcicine, and cis-dihydronarcicine, exhibit significant activity against Flavivirus and Bunyavirus in vitro [102].

#### 3.3.2. Mechanisms of Action Against Virus

##### Inhibiting Virus Replication

**Figure 3:**
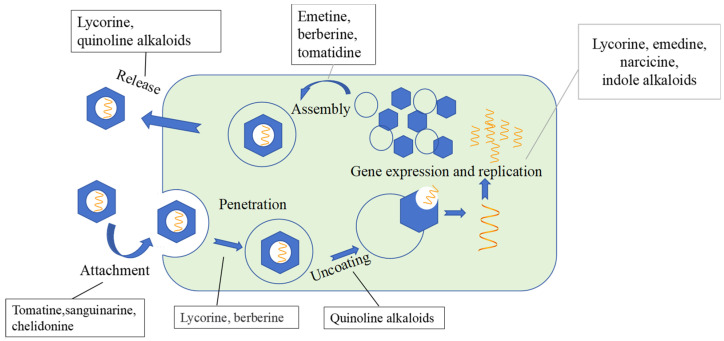
Mechanism of action of antiviral natural alkaloids along with their target sites.

As shown in Figure 3, many alkaloids exert their antiviral effects by targeting key genetic material and enzymes essential for the viral life cycle, disrupting their normal biological functions through various mechanisms. For example, during the early stages of viral replication, certain alkaloids have been shown to inhibit HSV-1 by blocking DNA synthesis [103]. Lycorine has been demonstrated to inhibit the RNA-dependent polymerase of both Zika and dengue viruses (DENV) [104]. Emedine has also shown significant inhibitory activity, with minimal cytotoxicity, by interfering with HIV reverse transcriptase and reducing HIV-1 infection by up to 80% [105]. Tomatine, a steroidal alkaloid derived from the stems and green leaves of tomato plants, has been found to exhibit antiviral activity against CHIKV infection. This activity is believed to result from its ability to interfere with the production of infectious viral particles, as well as its inhibition of viral attachment and replication following cell entry [106].

##### Inhibiting Viral Particle Assembly

Several alkaloids have been shown to inhibit protein synthesis across various viral families, with one proposed mechanism involving the disruption of ribosomal proteins, thereby hindering viral particle assembly. For example, emetine not only interferes with viral translation by degrading the 40S ribosomal protein S14 and blocking the synthesis of viral proteins in infected cells, but also inhibits HIV reverse transcriptase and viral polymerase, preventing the assembly of HIV particles [107]. Berberine chloride, a broad-spectrum alphavirus inhibitor, has been reported to disrupt the interaction and oligomerization of alphavirus Cp-gRNA, leading to defects in viral assembly [108]. Similarly, tomatidine exhibits potent antiviral activity by inhibiting the assembly of CHIKV virions [106].

## 4. Synergistic Effects of Alkaloids with Conventional Antimicrobial Agents

### 4.1. Overview of Studies Demonstrating the Synergistic Effects of Alkaloids with Antibiotics

In recent years, growing evidence has highlighted the synergistic effects between alkaloids and antibiotics, which can enhance the antibacterial efficacy of antibiotics, reduce the required drug dosage, minimize adverse side effects, and potentially reverse bacterial resistance [69]. Some studies have demonstrated that certain alkaloids can potentiate the antibacterial activity of β-lactam antibiotics (e.g., penicillins, cephalosporins). For example, methicillin-resistant *S. aureus* (MRSA) strains exhibit increased susceptibility to β-lactam antibiotics when combined with the alkaloid camptothecin, leading to enhanced bactericidal effects [109]. Other studies have shown that alkaloids can enhance the effectiveness of aminoglycoside antibiotics (e.g., gentamicin, neomycin) through synergistic interactions. For instance, the co-administration of the alkaloid cinchonidine with gentamicin against *P. aeruginosa* significantly increases the antibacterial activity of gentamicin, resulting in reduced minimum inhibitory concentrations (MICs) of the antibiotic [110].

Several studies have highlighted the synergistic effects of alkaloids when combined with macrolide antibiotics (e.g., erythromycin, azithromycin). For example, co-administration of the alkaloid quinine with erythromycin significantly enhances its antibacterial activity against *Chlamydia pneumoniae*, thereby reducing the required dosage of the antibiotic [111]. The synergistic interactions between alkaloids and antibiotics open up new possibilities for the development of antibacterial agents and offer alternative strategies for addressing antibiotic-resistant bacterial infections. However, further comprehensive studies and validation are needed to fully assess their clinical potential.

### 4.2. Examples of Alkaloids Exhibiting Synergistic Antimicrobial Effects

The literature provides numerous examples of alkaloids that exhibit synergistic effects with antimicrobial agents. A prominent example is berberine, a plant-derived alkaloid that has been extensively studied for its ability to enhance the efficacy of antibiotics. Evidence indicates that berberine can potentiate the antimicrobial activity of antibiotics such as penicillin and cephalosporins against various bacterial pathogens, including MRSA and multidrug-resistant *Escherichia coli* [112]. Similarly, sanguinarine has been shown to enhance the effects of antibiotics such as gentamicin and tetracycline, targeting a broad range of bacteria, including both Gram-positive and Gram-negative strains [113]. Additionally, alkaloids like quinine and cinchonidine have demonstrated synergistic effects with erythromycin and chloramphenicol against respiratory pathogens such as *Mycoplasma pneumoniae*.

Reserpine has been widely reported to potentiate the activity of fluoroquinolones and tetracyclines by inhibiting bacterial efflux pumps such as NorA, thereby restoring antibiotic susceptibility in resistant *S. aureus* strains [114]. Likewise, Piperine has demonstrated synergistic effects with antibiotics including ciprofloxacin and rifampicin, primarily through enhancing membrane permeability and inhibiting drug efflux mechanisms [115,116]. In addition, Harmine and related β-carboline alkaloids have been shown to enhance the antibacterial efficacy of β-lactam antibiotics and aminoglycosides against both Gram-positive and Gram-negative bacteria [117]. Furthermore, Capsaicin has been reported to synergize with conventional antibiotics by disrupting bacterial membranes and interfering with quorum sensing systems [118,119]. These additional examples, together with previously described alkaloids such as berberine and sanguinarine, further emphasize the broad spectrum of mechanisms—ranging from efflux pump inhibition to membrane perturbation—through which alkaloids can enhance antimicrobial efficacy.

### 4.3. Potential Use of Alkaloids as Adjuvants to Enhance Antimicrobial Efficacy

Alkaloids have shown promising potential as adjuvants to enhance the effectiveness of antimicrobial drugs [69,120]. One of the key benefits of alkaloids is their ability to enhance the activity of antimicrobial drugs through synergistic interactions. Research findings have demonstrated the potential of alkaloids in augmenting the effectiveness of antibiotics against a broad spectrum of bacterial pathogens, encompassing both Gram-positive and Gram-negative strains. This synergy can lead to more effective treatment outcomes and may help combat antibiotic resistance. Alkaloids support antimicrobial drugs by interfering with bacterial cell wall formation, disrupting cell membrane integrity, inhibiting protein synthesis, and affecting bacterial genetic material. By targeting bacterial physiology, they help overcome resistance mechanisms and enhance the efficacy of antibiotics.

For instance, berberine has been widely reported to exhibit synergistic effects with β-lactam antibiotics and fluoroquinolones against methicillin-resistant MRSA and multidrug-resistant *E. coli*, primarily through inhibition of efflux pumps and disruption of membrane function [121,122]. Similarly, sanguinarine has been shown to potentiate the activity of antibiotics such as gentamicin and tetracycline against both Gram-positive and Gram-negative bacteria by targeting bacterial membranes and intracellular processes [123]. In addition, reserpine acts as a well-known efflux pump inhibitor, significantly enhancing the activity of fluoroquinolones and tetracyclines against resistant *S. aureus* strains by blocking NorA-mediated drug efflux [124]. Piperine has also demonstrated synergistic effects with antibiotics such as ciprofloxacin and rifampicin, increasing intracellular drug accumulation and improving antibacterial efficacy [115]. These findings collectively highlight that alkaloids can function as resistance-modifying agents, restoring antibiotic susceptibility in resistant strains.

Another advantage of alkaloids is their broad spectrum of activity against a variety of bacterial species, making them useful for treating polymicrobial infections or multidrug-resistant bacteria. Alkaloids also show promise for treating bacterial, fungal, and parasitic infections, potentially leading to more effective regimens, shorter treatment durations, and reduced risk of relapse. However, the safety and toxicity profiles of alkaloids require careful evaluation, as some may be toxic at higher concentrations. Further research is needed to assess their safety and clinical utility as adjuvants in antimicrobial therapy.

## 5. Future Research Directions

### 5.1. Identification and Isolation of New Natural Alkaloids with Antimicrobial Activity

Future research directions in the field of alkaloids and antimicrobial activity involve the identification and isolation of new natural alkaloids with potent antimicrobial properties [9,125,126]. Systematic screening and bioassays can help pinpoint promising candidates for further investigation. On the other hand, understanding the mechanisms behind the antimicrobial effects of these alkaloids is crucial for identifying their mode of action and potential cellular targets. Molecular biology tools, such as gene expression analysis and proteomics, can provide insights into the cellular pathways affected by these compounds. Structure-activity relationship (SAR) studies will help optimize the chemical structure of alkaloids, guiding improvements in their pharmacological activity.

In recent years, several novel alkaloids with antimicrobial potential have been isolated from diverse natural sources, further enriching the chemical space for drug discovery. For example, new indole alkaloids isolated from Streptomyces species have demonstrated notable antibacterial activity against Gram-positive pathogens, including MRSA, with MIC values [127]. Similarly, a series of quinazoline and isoquinoline alkaloids obtained from marine-derived fungi and plants have shown inhibitory effects against both bacterial and fungal strains, highlighting the importance of marine microorganisms as a source of bioactive alkaloids [128]. In addition, newly identified β-carboline alkaloids from medicinal plants have exhibited antimicrobial and antibiofilm activities, suggesting their potential as lead compounds for overcoming microbial resistance [129]. Advances in chromatographic separation techniques and spectroscopic characterization, including high-resolution mass spectrometry (HR-MS) and nuclear magnetic resonance (NMR), have greatly facilitated the discovery of these novel structures. These recent findings underscore the continuing importance of natural product exploration, particularly from underexplored ecological niches, in identifying structurally diverse alkaloids with promising antimicrobial properties.

### 5.2. Structure-Activity Relationship Studies and Optimization of Natural Alkaloids

SAR studies will focus on investigating known natural alkaloids to understand how structural variations affect their antimicrobial activity [130]. Comprehensive biological activity evaluations can be conducted on alkaloids with different structural variations, including tests for inhibitory effects against bacteria, fungi, and parasites. MIC and IC_50_ will be determined, providing insights into the relationship between structure and activity. By comparing the biological activity data of different compounds, further insights into the relationship between structure and activity can be gained [131]. Optimized compounds will undergo further pharmacological research, including pharmacokinetic, toxicity, and safety assessments [132]. These endeavors aim to persistently refine the structural features of natural alkaloids, thereby advancing the development of antimicrobial agents with augmented efficacy and specificity. It is anticipated that these endeavors will furnish crucial support and direction for the exploration and innovation of novel antibiotics.

In addition to natural alkaloids, the synthesis and evaluation of their derivatives have emerged as a crucial strategy to enhance antimicrobial potency and overcome intrinsic limitations. Olleik *et al.* reported that specific berberine derivatives displayed markedly enhanced activity against Gram-positive bacteria, including resistant strains, highlighting the importance of structural optimization in improving pharmacological performance [133]. Similar strategies have been applied to other alkaloid classes, where derivatization leads to improved target specificity, reduced efflux susceptibility, and enhanced pharmacokinetic properties. Collectively, these findings emphasize that semi-synthetic modification of natural alkaloids represents a key direction in SAR studies, bridging the gap between natural product discovery and clinically relevant antimicrobial agents.

### 5.3. Structure–Activity–Toxicity Relationships of Natural Alkaloids

An important consideration in evaluating the therapeutic potential of natural alkaloids is the relationship between their antimicrobial activity and cytotoxicity. Increasing evidence suggests that different classes of alkaloids exhibit markedly distinct safety profiles when comparing MICs with cytotoxic concentrations (e.g., IC_50_ values) [125,134]. For instance, benzophenanthridine alkaloids such as Sanguinarine often display potent antimicrobial activity with MIC values in the low micromolar range; however, their cytotoxicity is similarly high (sub-micromolar to low micromolar IC_50_), resulting in a narrow therapeutic window [125,135]. In contrast, Berberine generally exhibit moderate antimicrobial activity (MICs typically ranging from 32 to 256 μg/mL), while showing comparatively lower cytotoxicity (IC_50_ often > 50–100 μM), suggesting a more favorable safety profile but limited potency as standalone agents [125,134]. Other alkaloids, including Dendrobine and Cyclovirobuxine D, tend to exhibit both weak antimicrobial activity (high MIC values) and low cytotoxicity, indicating limited direct therapeutic relevance but potential as scaffolds for structural optimization [136].

From a pharmacological perspective, the selectivity index (SI), defined as the ratio of cytotoxic concentration to antimicrobial activity (e.g., IC_50_/MIC), provides a useful metric for evaluating the balance between efficacy and safety [135]. Many naturally occurring alkaloids exhibit low SI values due to overlapping antimicrobial and cytotoxic concentration ranges, which may restrict their clinical application. These findings highlight the importance of optimizing the SI to achieve a balance between efficacy and safety, reinforcing the need for structural modification and combination strategies in alkaloid-based drug development.

### 5.4. Evaluation of the Safety and Efficacy of Natural Alkaloids in Clinical Trials

The process of evaluating the safety and efficacy of natural alkaloids in medical trials constitutes a pivotal step in their development as potential antimicrobial agents. This process involves rigorous testing in human subjects to assess the safety profile, pharmacokinetics, pharmacodynamics, and therapeutic efficacy of these compounds [137]. Clinical trials also measure the efficacy of alkaloids in treating infections by evaluating microbial load reduction, symptom resolution, and overall patient outcomes. Comparative studies may assess their effectiveness against standard antimicrobial therapies. Pharmacokinetic research explores how alkaloids are absorbed, distributed, metabolized, and eliminated, while pharmacodynamics studies optimize dosing by examining the relationship between drug concentration and microbial response [138].

Representative alkaloids that have undergone clinical or translational evaluation further illustrate the balance between efficacy and safety. For instance in Table 2, Berberine has demonstrated favorable safety and moderate efficacy in clinical settings, particularly as an adjunct agent, although its relatively low bioavailability limits systemic antimicrobial applications [139]. In contrast, Colchicine exemplifies compounds with a narrow therapeutic window, where clinical efficacy is accompanied by dose-dependent toxicity, necessitating careful monitoring [140]. Similarly, Reserpine highlights safety limitations due to central nervous system side effects, which have restricted its broader therapeutic use despite pharmacological activity [114]. More clinically successful examples include Quinine and Vincristine, which demonstrate that alkaloids can achieve significant therapeutic efficacy when appropriately optimized, although toxicity (e.g., cardiotoxicity or neurotoxicity) remains a key concern [141]. In contrast, Capsaicin illustrates a favorable safety profile in topical applications, suggesting that alternative delivery strategies may effectively mitigate systemic toxicity [142].

Overall, these examples underscore that the clinical translation of natural alkaloids is often constrained by a trade-off between efficacy and toxicity. Therefore, future research should focus on improving pharmacokinetic properties, reducing toxicity, and exploring combination or adjuvant strategies to expand the therapeutic window and enhance clinical applicability.

### 5.5. Developing Combination Therapy

Developing combination therapy involves the strategic use of multiple antimicrobial agents, including natural alkaloids, to enhance the effectiveness of treatment and overcome antimicrobial resistance. This approach aims to exploit synergistic interactions between different agents to achieve greater antimicrobial activity, reduce the risk of resistance development, and improve treatment outcomes [146,147]. Combination therapy could enhance the handling of infectious diseases and combat antimicrobial resistance, offering new strategies for effective antimicrobial treatment.

Recent preclinical studies have provided substantial evidence supporting the synergistic potential of alkaloid–antibiotic combinations. For example, Berberine has been widely shown to enhance the activity of β-lactams and fluoroquinolones against MRSA and multidrug-resistant *E. coli*, primarily through inhibition of efflux pumps and disruption of membrane integrity [122,148]. Similarly, Sanguinarine has demonstrated synergistic effects with antibiotics such as gentamicin and tetracycline, enhancing antibacterial activity against both Gram-positive and Gram-negative bacteria by targeting bacterial membranes and intracellular processes [123]. In addition, Reserpine is a well-characterized efflux pump inhibitor that significantly potentiates the efficacy of fluoroquinolones and tetracyclines by blocking NorA-mediated drug efflux in resistant *S. aureus* strains [124]. Furthermore, Piperine has been reported to enhance the intracellular accumulation of antibiotics such as ciprofloxacin and rifampicin, thereby improving antibacterial efficacy [115]. These findings highlight multiple mechanisms underlying synergy, including efflux pump inhibition, membrane disruption, and increased intracellular drug retention.

Beyond antibacterial applications, alkaloid-based combination strategies have also shown promise in antifungal therapy. Certain alkaloids have been reported to enhance the activity of azole antifungals by interfering with ergosterol biosynthesis pathways or increasing membrane permeability, suggesting potential relevance for targeting enzymes such as CYP51. These findings are particularly important in the context of emerging antifungal resistance and highlight the potential of alkaloids as adjuvants in antifungal regimens.

Emerging clinical and translational studies further support the feasibility of combination strategies. For instance, Berberine has been evaluated as an adjunct therapy in clinical settings, demonstrating improved therapeutic outcomes when combined with conventional drugs, particularly in infections and metabolic disorders [139]. Additionally, Colchicine has been successfully used in combination regimens for inflammatory conditions, illustrating the broader applicability of alkaloid-based combination strategies in modulating disease pathways [140]. Although direct clinical evidence for antimicrobial combination therapy involving alkaloids remains limited, these studies provide a strong foundation for future clinical translation.

Despite these promising advances, several challenges remain. These include the need for standardized evaluation methods for synergy (e.g., checkerboard assays and time–kill studies), potential drug–drug interactions, and safety concerns associated with alkaloid toxicity. Therefore, future research should focus on well-designed *in vivo* studies and clinical trials to validate the efficacy and safety of alkaloid-based combination therapies. Overall, the integration of natural alkaloids into combination therapy represents a promising and innovative strategy to address the growing challenge of antimicrobial resistance.

### 5.6. Alkaloids as Resistance-Modifying Agents Against Drug-Resistant Pathogens

A particularly important aspect of alkaloid research is their activity against antimicrobial-resistant pathogens. Increasing evidence indicates that many natural alkaloids exhibit significant inhibitory effects on drug-resistant bacteria, including MRSA, vancomycin-resistant enterococci (VRE), and multidrug-resistant Gram-negative strains [112,149]. Berberine has been reported to inhibit the growth of MRSA and resistant *E. coli*, although its activity is often enhanced in combination with efflux pump inhibitors or antibiotics [112]. Similarly, Sanguinarine exhibits potent antibacterial activity against resistant Gram-positive bacteria through membrane disruption and DNA interaction [150]. In addition, Reserpine plays a crucial role in reversing antibiotic resistance by inhibiting bacterial efflux systems such as NorA, thereby restoring susceptibility to conventional antibiotics [149].

Mechanistically, alkaloids target multiple pathways associated with resistance, including inhibition of efflux pumps, disruption of biofilm formation, alteration of membrane permeability, and interference with resistance-related gene expression [149,150]. These multitarget effects reduce the likelihood of resistance development and make alkaloids particularly valuable as resistance-modifying agents. Moreover, recent studies have shown that certain alkaloids can effectively disrupt biofilms formed by resistant bacteria, further enhancing their therapeutic potential [112].

Despite these promising findings, most evidence remains limited to *in vitro* and preclinical studies, and standardized evaluation against resistant clinical isolates is still lacking. Therefore, future research should prioritize clinically relevant resistant strains, *in vivo* validation, and mechanistic studies to fully elucidate the role of alkaloids in combating antimicrobial resistance.

## 6. Conclusions

Natural alkaloids represent a chemically diverse source of bioactive compounds with demonstrated antimicrobial activity against bacteria, fungi, and parasites. However, despite extensive *in vitro* evidence supporting mechanisms such as membrane disruption, inhibition of protein synthesis, and interference with nucleic acid function, their clinical translation remains limited. Recent studies highlighting additional anti-inflammatory and immunomodulatory effects suggest that alkaloids may exert therapeutic benefits beyond direct antimicrobial action, particularly through modulation of host–pathogen interactions.

From a pharmacological perspective, key advances in structure–activity relationship studies have identified features such as planar aromaticity and cationic centers as critical determinants of activity. Compounds such as Berberine and Sanguinarine exemplify multitarget antimicrobial mechanisms, whereas Reserpine highlights an alternative paradigm as a resistance-modifying agent. Nevertheless, these pharmacological advantages are often offset by significant limitations, including low bioavailability, non-specific mechanisms, and potential cytotoxicity at therapeutically relevant concentrations.

Importantly, the majority of current evidence is derived from *in vitro* or preclinical studies, with a clear lack of well-designed clinical trials validating antimicrobial efficacy. In addition, variability in experimental methods, absence of standardized synergy evaluation, and insufficient pharmacokinetic data further hinder translational progress. Resistance to certain alkaloids has also been reported, raising concerns about their long-term effectiveness.

Therefore, future research should move beyond descriptive antimicrobial screening toward mechanism-driven and clinically oriented studies, including rigorous *in vivo* validation, optimization of pharmacokinetic properties, and rational design of combination therapies. Integration of emerging technologies, such as AI-assisted drug discovery, may accelerate this process but must be coupled with experimental validation.

In conclusion, while natural alkaloids hold promise as antimicrobial agents or adjuvants, their true clinical value will depend on overcoming current limitations and establishing clear evidence of safety, efficacy, and mechanistic specificity.

## Figures and Tables

**Table 1:** Representative natural alkaloids with a wide range of biological activities.

Number	Name	Chemical Structure	Sources	Categorization	Biological Activities	Refs.
1	Acronycine	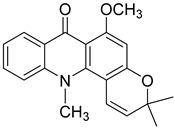	*Acronychia pedunculata* L.	Acridone alkaloid	Anticancer; no antimicrobial activities.	[29,30]
2	Berberine	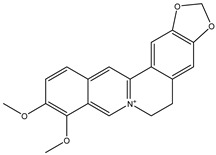	*Coptis chinensis* Franch.	Quaternary ammonium isoquinoline alkaloid	Berberine exhibited antifungal effects on *C. albicans* and *C. neoformans*, with IC_50_ of 30.92 to 50.93 µg/mL; It also displayed activity against *T. mentagrophytes*, with an MIC value of 64 µg/mL.	[31]
3	Camptothecin	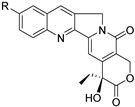	*Camptotheca*	Quinoline alkaloid	DNA topoisomerase I inhibitor; Camptothecin inhibits yeast topoisomerases with an IC_50_ of 17.8 µM.	[32,33]
4	Cephalotaxine	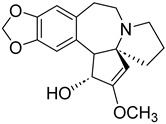	*Cephalotaxus*	*Cephalotaxus* Alkaloid	Anticancer; Cephalotaxine exhibits negligible antimicrobial activity.	[34]
5	Colchicine	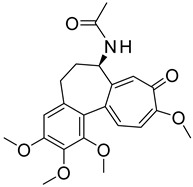	*Colchicum autumnale* L.	Zhufenone alkaloid	Antimitotic activity; Anti-*Aureobasidium pullulans* (MFC:1 μg/mL).	[35,36]
6	Codonopsine	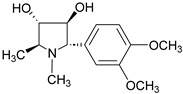	*Codonopsis clematidea* (Schrenk) C.B.Clarke	Pyrrolidine alkaloid	Antihypertensive effects (regulates vascular tone); Poor activity against Gram-negative pathogens like *Pseudomonas aeruginosa* and *Klebsiella pneumoniae* (MIC ≥ 512 µg/mL).	[37,38]
7	Cyclovirobuxine-D	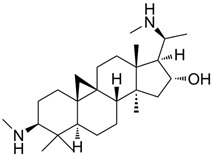	*Buxus sinica* L.	*Buxus* alkaloid	Calcium inhibitor; Cyclovirobuxine D exhibits weak antimicrobial activity.	[39]
8	Dendrobine	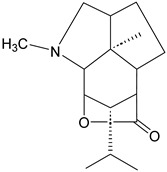	*D* *endrobium*	Terpenoid alkaloid	Relieve pain and reduce fever; Dendrobine exhibits weak antimicrobial activities.	[40]
9	Emetine	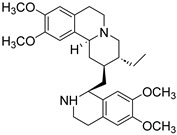	*Rubiaceae*	Isoquinoline alkaloid	Antivirals and emetic effects; Anti-Herpesviruses (EC_50_ ≤ 0.056 μM).	[41,42]
10	Leonurine	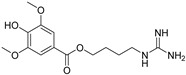	*Leonurus sibiricus* L.	Phenylpropanoid alkaloid	Antitumor, anti-inflammatory, antiapoptotic effects; Anti-*Mycobacterium tuberculosis* (MIC: 80 μg/mL).	[43,44,45,46]
11	Matrine	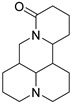	*Sophora flavescens* L. and *Radix sophorae tonkinensis* L.	Quinolizidine alkaloid	Anti-cancer; Anti-*S. mutans* (MIC: 10 mg/mL).	[47,48,49,50]
12	Monocrotaline	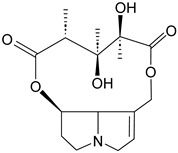	*Crotalaria genus* L.	Pyrrolizidine alkaloid	Hepatotoxicity;Anti-*T. vaginalis* (MIC: 1 mg/mL).	[51,52,53]
13	Piperine	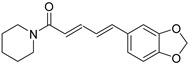	*Piper nigrum* L.	Piperidine alkaloid	Broad-spectrum anticonvulsant effects; Anti-*S. aureus* (IC_50_: 59 µg/mL).	[54,55,56]
14	Papaverine	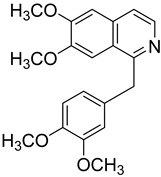	*Papaveraceae*	Benzylisoquinoline Alkaloid	Vasodilation;Inactive against *E. coli* and MRSA.	[57,58,59]
15	Pseudoephedrine	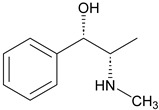	*Ephedra*	Phenethylamine alkaloid	Sympathomimetic effects; Anti-*E. coli* (>512 µg/mL, no activity).	[60]
16	Reserpine	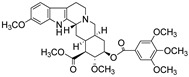	*Palea steindachneri* L.	Indole alkaloid	Antidepressant and antihypertensive effects; Reserpine exhibits weak intrinsic antimicrobial activity (MIC typically > 128 μg/mL).	[61,62]
17	Scopolamine	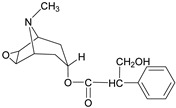	*Atropa belladonna* L.	Tropane alkaloid	Nonselective muscarinic antagonist; Anti-*Streptococcus pyogenes* (MIC: 8 mg/mL); Anti-*Shigella dysenteriae* (MIC: 2 mg/mL).	[63,64,65]
18	Securinine	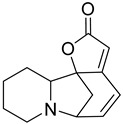	*Flueggea*	Indolizidine alkaloid	Topoisomerase I (Topo I) Inhibitors; Multispecies biofilm (*L. monocytogenes*, *S. Typhimurium*, *P. aeruginosa*) eradication: 78.9–99.8% at 25 mg/mL.	[66,67]
19	Salsoline	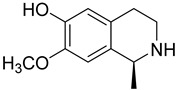	*Equisetum arvense* L.	Isoquinoline alkaloid	Antihypertensive effects; Salsoline itself exhibits negligible antimicrobial activity (MIC typically > 200 μg/mL or inactive).	[68]

**Table 2:** Clinical evaluation of safety and efficacy of representative natural alkaloids.

Alkaloid	Clinical Application	Study Type	Efficacy Outcomes	Safety Profile	Key Findings	Refs.
Berberine	Metabolic disorders, infections	Randomized clinical trials (RCTs)	Improved glycemic control; adjunct antimicrobial effects	Generally well tolerated; mild GI effects	Demonstrates multi-target activity; potential as adjunct therapy	[139]
Homoharringtonine	Leukemia (AML, CML)	Phase II/III clinical trials	Significant anti-leukemic efficacy	Hematological toxicity manageable	Approved in some regions; strong clinical validation	[143]
Colchicine	Gout, cardiovascular inflammation	Large-scale RCTs	Reduced inflammation and cardiovascular events	Narrow therapeutic index; GI and hematologic toxicity	Well-established drug; dose-dependent toxicity	[140]
Reserpine	Hypertension	Clinical use (historical)	Effective blood pressure reduction	CNS side effects (depression, sedation)	Limited modern use due to safety concerns	[144]
Quinine	Malaria	Clinical trials and long-term use	Effective antimalarial activity	Cinchonism, cardiotoxicity at high doses	Still used in resistant malaria cases	[141]
Vincristine	Cancer (leukemia, lymphoma)	Phase III clinical trials	High efficacy in chemotherapy regimens	Neurotoxicity (dose-limiting)	Widely used in combination therapy	[145]
Capsaicin	Neuropathic pain	Clinical trials (topical)	Significant pain reduction	Local irritation; minimal systemic toxicity	Safe for topical application	[142]

## Data Availability

Not applicable.
